# HEALTH BEHAVIORS ASSOCIATED WITH SUBJECTIVE COGNITIVE DECLINE IN OLDER ADULTS

**DOI:** 10.1093/geroni/igz038.3326

**Published:** 2019-11-08

**Authors:** Kristi M Wisniewski, Elizabeth M Zelinski

**Affiliations:** 1 University of Southern California, Los Angeles, California, United States; 2 Davis School of Gerontology, University of Southern California, Los Angeles, California, United States

## Abstract

Participation in risky health behaviors can increase the potential for cognitive decline. Smoking, alcohol consumption, and minimal physical activity are modifiable risk factors associated with worse performance on cognitive assessments; however, the relationship between subjective cognitive decline (SCD) and risky practices has not been assessed. As a potential early indicator of cognitive impairment, SCD may serve as a screening measure for dementia. The Behavioral Risk Factor Surveillance System is an annual, self-reported telephone survey of Americans that includes fifteen core and twenty-five optional sections. The present study included Behavioral Risk Factor Surveillance System participants age 45 or older who completed the core and cognitive decline modules in 2015 (n=147,243). Roughly 11% of participants endorsed worsening memory in the previous year. Logistic regression examined the impact of smoking, drinking, and inactivity on self-reported cognitive decline. Current or former smokers had greater odds of endorsing cognitive decline compared to those who never smoked (OR=1.4; 95% CI: 1.27-1.52). Individuals who consumed at least one alcoholic beverage in the previous month had lower SCD odds compared to non-drinkers (OR=0.8; 95% CI: 0.72-0.87). Respondents who engaged in little to no physical activity had greater odds of endorsing cognitive decline compared to active respondents (OR=1.4; 95% CI: 1.31-1.57). Individuals who endorsed cognitive decline engaged in unhealthy habits such as smoking or inactive lifestyles; however, low to moderate alcohol consumption may be beneficial for cognitive functioning.

